# Video-based e-learning program for schoolteachers to support children of parents with mental illness: a cluster randomized trial

**DOI:** 10.1186/s12889-023-15426-z

**Published:** 2023-03-18

**Authors:** Masako Kageyama, Atsunori Matsushita, Ayuna Kobayashi, Taku Sakamoto, Yasuhiro Endo, Setsuko Sakae, Keiko Koide, Ryotaro Saita, Hiyuka Kosaka, Satoko Iga, Keiko Yokoyama

**Affiliations:** 1 Osaka University Institute of Advanced Co-Creation Studies, 1-7 Yamadaoka, Suita, Osaka 565-0871 Japan; 2 Osaka City Tamagawa Primary School, 2-13-16 Tamagawa, Fukushima-Ku, Osaka, 553-0004 Japan; 3 KODOMO-PEER Tonoxbuilding, 3-5-1 Hirata, Ichikawa, Chiba 272-0031 Japan; 4 Kokubunji 9Th Elementary School, 4-12-6 Nishi-Koigakubo, Kokunji, Tokyo, 185-0013 Japan; 5 Department of Social Design, Faculty of Sociology, St. Andrew’s University, 1-1 Manabino, Izumi, Osaka 594-1198 Japan; 6 Department of Health Promotion Science, Osaka University Graduate School of Medicine, 1-7 Yamadaoka, Suita, Osaka 565-0871 Japan; 7 Department of Medical Innovation, Osaka University Hospital, 2-2 Yamadaoka, Suita, Osaka 565-0871 Japan; 8 Department of Traumatology and Acute Critical Medicine, Osaka University Hospital, 2-15 Yamadaoka, Suita, Osaka 565-0871 Japan; 9 Department of Nursing, Faculty of Nursing, Josai International University, 1 Gumyo, Togane-City, Chiba-Pref 283-8555 Japan; 10 Department of Nursing, Faculty of Nursing, Yokohama Soei University, 1Miho-Cho, Yokohama City, Kanagawa 226-0015 Japan

**Keywords:** Mental illness, Children, Parental mental illness, Schoolteachers, Professional development, Mental health literacy, E-learning program, Cluster RCT

## Abstract

**Background:**

Some children of parents with mental illness need support. This study aimed to develop and test the effectiveness of an e-learning program for training elementary schoolteachers to support children of parents with mental illness.

**Methods:**

The program, which included a 30-min video-based e-learning program, aimed to help schoolteachers gain basic knowledge about mental illness and children of parents with mental illness, recognize children in need of support, and gain confidence in supporting them. A school-based cluster randomized controlled trial was conducted, and the schools were randomly divided into intervention and control groups. The teachers at these schools signed up for the program and participated individually. The outcome measures for the schoolteachers were evaluated at three time points: baseline (T1), post (T2), and one month later (T3). Along with the Sense of Coping Difficulty subscale (primary outcome measure), the following self-developed outcome measures were used: actual behaviors and attitude toward supporting children, knowledge, and self-assessment of program goals achievement. The Sense of Coping Difficulty subscale results at T3 were compared between the groups. Effectiveness over time was assessed for all the outcome measures. The interaction between baseline and intervention effects on the Sense of Coping Difficulty subscale was analyzed. As a part of the process evaluation, open-ended text responses were analyzed qualitatively.

**Results:**

Baseline responses were collected from 87 participants in the intervention group and 84 in the control group. The total score of the Sense of Coping Difficulty subscale at T3 was significantly lower in the intervention group than in the control group (*p* = 0.007). Over time, a significant effect was observed on the Sense of Coping Difficulty subscale, actual behavior, knowledge of onset timing and probability of onset, and achievement of all program goals. Exploratory analysis was particularly effective for those who encountered a high level of difficulty in supporting children. The participants’ text responses indicated that they planned to look carefully at children's backgrounds and stay close to them in the future.

**Conclusions:**

The program was effective for schoolteachers in supporting children of parents with mental illness.

**Trial registration:**

UMIN000045483; 14/09/2021.

## Background

Mental illness is an important public health issue affecting many people, with one in five Japanese individuals reportedly being affected during their lifetime [1]. The percentage of people with mental illness who become parents is not different from that of people without mental illness [2, 3]. It is estimated that 15–23% of children live with parents affected by mental illness [4]. Qualitative studies that have examined the experiences of children of parents with mental illness have reported that they experience difficulties related to coping, the burden of care roles, stigma, and isolation [5–8] and have to deal with long-term effects even after growing up [9]. Moreover, children of a parent with mental illness are 2.5 times more likely to develop mental illness themselves [10]. Not all children of parents with mental illness require support, but some do [11, 12].

In Japan, it has only been about 15 years since the existence of children raised by parents with mental illness became known to society [13]. In recent years, issues related to young carers have received a great deal of attention in the press, and social concern has increased; furthermore, the national government has implemented some initiatives to establish a counseling system to help them address this issue [14]. A national survey on young carers was conducted among middle and high school students in 2020; among students caring for their parents, physical illness was found to be the most common reason parents required care, followed by mental illness [15]. According to a survey conducted among adult children of parents with mental illness, more than 90% recalled and believed that their parents experienced mental illness before or during their own elementary school years [16]. Parents with mental illness are often “hidden” as a family secret due to the fear of stigma, and this hampers the identification of children who may require support [5, 6, 8].

Even if the children themselves are caring for their parents or struggling with family matters, they are often reluctant to seek help because of stigma [17]. A similar situation exists in Japan; adult children of parents with mental illness recalled that, when they were in elementary school, more than 90% did not consult their schoolteachers [16]. A survey of young cares in elementary school showed that family and friends were the most common people that the young carers consulted; however, the most common professional individual they consulted was found to be that of schoolteachers [18]. Not all children need support, but identifying the children in need of support is a starting point for support provision [19]. Schoolteachers are expected to act like adults who can identify children in need of support [19]. However, less than half of all elementary schools in a national survey identified young carers in need of support; this indicated that schoolteachers often found it difficult to identify such children because of difficulties in understanding the children’s home situations [18]. A previous study reported that schoolteachers need knowledge about the signs to look for and information about how to support the children of parents with mental illness, that schools play an important role in supporting children, and that proper training is required for recognizing and supporting children who need help [20]. The study recommended some strategies to develop individual relationships with at risk children and to promote school wide programs [20].

Programs for improving schoolteachers’ mental health literacy have already been developed; many of these have induced improvements in schoolteachers’ knowledge, attitudes, and confidence in supporting the mental illness of students [21]. Mental health literacy has been defined as “knowledge and beliefs about mental disorders, which aid their recognition, management, or prevention” [22]. Mental health literacy includes the following components: prevention of mental disorders, early detection, help-seeking, self-help strategies, and first aid skills to support others [23]. Although mental health literacy focuses on prevention and early treatment, the support provision situation is different when a parent has a mental illness, as such an illness may already be chronic [24]. Mental health literacy education has some useful aspects (e.g., general knowledge regarding mental illness); however, it is often difficult to apply mental health literacy education programs directly to children caring for parents with mental illness [24]. Therefore, programs specifically designed to train schoolteachers in supporting children of parents with mental illness are necessary; however, there have been no reports on such programs.

In Japan, 50-min video material has been developed for schoolteachers to improve their mental health literacy [25]. Specifically, it aims to increase their knowledge, decrease the stigma, and increase their intention to support students with mental illness [25]. A large-scale survey of Japanese elementary and junior high schools found that schoolteachers work overtime for an average of 16.4 h per week on weekdays; furthermore, their longer overtime hours can lead to higher psychological stress [26]. Therefore, shorter instructional videos are required. Moreover, e-learning does not require them to devote more hours, as they can watch the videos while commuting or during gap time.

This program aimed to help schoolteachers gain basic knowledge about mental illness and children of parents with mental illness, recognize children in need of support, and gain confidence in supporting them. In mental health literacy education aimed at schoolteachers, their knowledge regarding mental illness and confidence in supporting students in need of support often indicate a program’s effectiveness [21]. In addition to the effectiveness, we have added the knowledge about children of parents with mental illness and behaviors to recognize children in need of support because they are not easily consulted to adults [16]. This study aimed to develop and test the effectiveness of a video-based e-learning program for training elementary schoolteachers to support children of parents with mental illness.

## Methods

### Design

A school-based cluster randomized controlled trial (RCT) was conducted to test the effectiveness of the program. Contamination of the information provided across groups within the same school may have occurred. Therefore, the RCTs of schoolteachers could use schools as clusters [21]. The schools were randomly divided into intervention and control groups. After the schools signed up for the study, the schoolteachers at these schools signed up individually.

The program, which was offered online on an individual basis, could only be watched by the intervention group. The outcome measures were evaluated using a self-administered web survey by comparing the two groups at three time points: baseline (T1), post (T2), and one month later (T3). Ethical considerations allowed the control group to participate in the same program after the final T3 response. No major changes were made to the methods after trial commencement. The trial findings were reported in a subsequent publication in accordance with the Consolidated Standards of Reporting Trials (CONSORT).

### Study participants

Requests for research participation were sent to 322 public elementary schools in three Japanese prefectures. Only schools that were willing to cooperate were included in the study.

A maximum of 10 schoolteachers participated from each school. The inclusion criterion was full-time schoolteachers, and the exclusion criteria were difficulty in understanding Japanese and being unable to conduct web surveys or operate video watching. The sample size was determined with reference to 50-min video programs for improving schoolteachers’ mental health literacy in Japan [21, 25]. The effect size was 0.55–0.97. Because the program of this study was shorter in duration, we assumed an effect size of 0.4. With the estimated number of schools being 30, α = 0.05, β = 0.20, and ICC = 0.02, this would yield a required study population of 221 participants. The sample size was set at 244, with a dropout rate of approximately 10%.

### Program development

The program was called the WATASHI-KOKO (“We are here” in Japanese) PROGRAM. It involved a 30-min video-based e-learning program for elementary schoolteachers, which was designed to help them support children of parents with mental illness. It aimed to help schoolteachers gain basic knowledge about mental illness and children of parents with mental illness (Purpose 1), recognize children in need of support (Purpose 2), and gain confidence in supporting them (Purpose 3).

The program was created with the support of “KODOMO-PEER” (“children’s companions” in Japanese), which is the largest self-help group for adult children of parents with mental illness in Japan. The program content was created based on a childhood needs survey that received responses from adult children of parents with mental illness [16]. First, two core members of KODOMO-PEER and the first author of this current paper created a draft program content for a year based on the survey results [16]. Next, 13 core members of KODOMO-PEER and seven multidisciplinary professionals, including schoolteachers, a public health nurse, psychiatric nurses, and social workers, revised the draft program content through discussions held in four meetings every three weeks (8 h in total) and via email. Most of their feedback was reflected in the video content.

To gain basic knowledge about mental illness (Purpose 1), the program content was created with reference to previous research on a mental health literacy education program for schoolteachers [25, 27]. The program included types of mental illness and their main symptoms; furthermore, it reflected the fact that mental illness affects 1 in 5 people in their lifetime and that half of those affected by mental illness develop the illness by their mid-teens (No.3 of Table 1).

**Table 1:** Components of the program

No		Title	Concrete contents
1	video	Gaining attention	Showing children in need of support, asking "Have you ever met a child like this?," and indicating percentage of children of parents with mental illness and percentage of such children not consulted
2	video	Program goals	Viewing target subject, program goals
3	video	Parental mental illness and children's lives	Types of mental illnesses and number of patients, children's life experiences, young carer roles, and impact on children's health
4	video	Until the audience recognize a child in need of assistance	Reasons why it is difficult to notice signs that those around schoolteachers can notice
5	video	After recognizing the child	Supporting needs of such children through school teachers, and creating an environment where schoolteachers feel comfortable asking for help at school
6	video	Message from an adult child of parents with mental illness	The experiences of a woman who was raised by a mother suffering from schizophrenia, her experiences with schoolteachers, and her message to schoolteachers
7		Self-assessing learning outcomes	Write what participants learned and how participants will apply this knowledge to their own future performance

To gain basic knowledge about children of parents with mental illness (Purpose 1), the program content was created based on a survey that received responses from adult children of parents with mental illness [16]. The survey found that, when in elementary school, 78% had experienced “anxious feelings,” 51% experienced “physical and mental problems of their own,” 63% experienced “constant fights between adults at home,” 51% experienced “attacks from parents,” 32% experienced “lack of laundry and cleaning,” 19% experienced “insufficient provision of food,” and 79% reported that they experienced difficulties with their parents. The program thus included children’s life situations and feelings as specific examples, along with the survey findings (No. 3 of Table 1).

To help schoolteachers recognize children in need of support (Purpose 2), the program content was developed based on the survey that received responses from adult children of parents with mental illness [16]. In the survey, 92% of respondents indicated that they did not consult with their schoolteachers during elementary school. Thus, the following signs were highlighted as being important for recognizing a child in need of support: no parent shows up for parent-teacher conferences or class visits, a child is often late or absent from class, a child forgets many things, a child has trouble concentrating on his/her studies, or a child has poor grades. The program included these signs (No. 4 of Table 1).

To help schoolteachers gain confidence in supporting children in need of support (Purpose 3), the program content was developed based on the survey [16]. In an open-ended text response part of the survey, where the adult children of parents with mental illness were asked to state what they appreciated about their schoolteachers, the following responses were provided: staying close to the children, talking to them, listening to them, being kind and friendly, responding to their parents when they were not feeling well, allowing them to go to the school nurse’s office, and being understanding to them and their families. Next, past research has shown that many children of parents with mental illness report a lack of trustworthy adults in their childhood, which remains a barrier to building trusting relationships even in adulthood [7]. The program, therefore, included information about how children often want their schoolteachers to treat them based on the survey responses and how they can be trustworthy adults for children. Moreover, based on the opinions of the schoolteachers who were research members, the program included the following suggestions for schoolteachers to help them deal with a parental mental illness situation at school: consult with other schoolteachers instead of worrying by themself, create an environment where it is easy to consult other schoolteachers, and persistently involve oneself in the situation without giving up—even if the relationship with the parents does not improve. The program included information about how schoolteachers could deal with the situation at school. Furthermore, it discussed life support services available for people with mental illness and provided examples (No. 5 of Table 1). For instance, the school discussed how to support, including the help of school social workers, and introduced home nursing in collaboration with other agencies.

Theoretically, the program content was also based on a four-tiered approach for delivering professional development programs related to parental mental disorders [28]. In this approach, the foundation of professional learning lies in reducing the public stigma that professionals often experience toward persons with mental illness. Because narrative messages have been reported to be effective as anti-stigma messages [29], stories of adult children of parents with mental illness were presented in this program (No. 3–6 of Table 1). The next step in the approach is to raise professional awareness of the needs of both children and parents. This current program included children’s needs (No.3 of Table 1), with reference to a survey of childhood experiences in schools [16]. The third step in the approach includes content specific to professionals. Accordingly, the current program included content focused on schoolteachers (No. 5 of Table 1).

In accordance with the learning process, the content structure of the program was based on the Transtheoretical Model (TTM) of health behavior change by Prochaska et al. [30] and Gagné’s instruction [31]. The TTM model divides the process of changing behaviors into stages and uses effective approaches for each stage [30]. The stages are pre-contemplation, contemplation, preparation, action, maintenance, and termination [30]. Since the existence of children of parents with mental illness is not well recognized in Japanese society [13], we decided to focus on the stages of pre-contemplation and contemplation and included consciousness-raising, emotional arousal, and self-re-evaluation as effective approaches. To raise awareness about children of parents with mental illness, we included knowledge and objective information about the relevant illnesses and the discussed children (No.3 of Table 1); for emotional arousal, we included storytelling from a grown child (No.6 of Table 1). For self-re-evaluation, we asked the participants the following question: “Have you ever met a child like this?” (No. 1 of Table 1).

Gagné’s instruction is an effective process when teaching others [31]. The learning process is, first, about getting attention (No. 1 of Table 1). This program informed schoolteachers about children in need of support, asking them, “Have you ever met a child like this?” and indicating the percentage of children of parents with mental illness as well as the percentage of such children who did not consult others. The next step involved informing the schoolteachers about the program goals (No. 2 of Table 1). Then, the schoolteachers were provided with new knowledge and ways to support the children (No. 3–5 of Table 1). The feedback process introduced the child’s narrative story; this included experiences the child had had with schoolteachers (No. 6 of Table 1). Finally, as part of the process of assessing learning outcomes, after watching the video, the participants were asked what they had learned and how they would apply the acquired knowledge to their future performance (No. 7 of Table 1).

The specifics of the program are shown in Table 1.

### Outcome measures

The outcomes were measured at the individual level. The questionnaire was developed by multidisciplinary experts who were research members of this project. The pre-test was conducted among 18 elementary schoolteachers. After watching the video of the program, they were asked to answer the questionnaire and also asked to indicate any points where they had found it difficult to understand sentences or words. Consequently, a few points were raised by the schoolteachers, and some of the wording was corrected.

#### Confidence in supporting children

The Sense of Coping Difficulty subscale was used to assess confidence in supporting children (Purpose 3 of the program). The Sense of Coping Difficulty/Possibility Scale refers to analogous concepts of self-efficacy, which is the expectation that professionals can deal with difficult situations [32, 33]. The scale has been tested for reliability and validity and consists of two subscales: Sense of Coping Difficulty and Sense of Coping Possibility [32, 33]. In this study, the total score of the Sense of Coping Difficulty subscale at one-month post-intervention (T3) as a primary outcome measure was calculated. The Sense of Coping Difficulty subscale consists of five items. This study asked about children and parents with mental illnesses. The five items were: "It is difficult to intervene because I feel that intervening will isolate children or parents from the community," "I do not know how to get involved if the children or parents refuse to meet with me," "I do not know how to get involved because the children or parents have special problems," "It is difficult to change children or parents," and "I do not know how to respond if I want to support but the children or parents will not respond.” For each item, the total score was given as "not at all disagree" (1), "disagree" (2), "agree" (3), or "agree very much" (4). Higher scores mean that they felt more challenged in supporting the children of parents with mental illness.

#### Actual behaviors and attitude

Three self-developed questions were used at T1 and T3 in order to assess the effects of program Purposes 2 and 3 on behaviors and attitudes. The program introduced the life situations of children in need of support and highlighted certain signs to look out for when identifying such children. The first question asked schoolteachers whether, over the past month, they had looked at children with the perspective that they could be children in need of support. The second question asked schoolteachers whether, over the past month, they had recognized the presence of a child(ren) in need of support. The program recommended that schoolteachers who had recognized a child in need of support first consult with other teachers in the school instead of worrying about the situation by themselves. The third question asked schoolteachers whether, over the past month, they had consulted other schoolteachers within the school about a child(ren) in need of support. The schoolteachers responded to these questions using “yes” or “no” options.

#### Knowledge

Knowledge questions, with responses that could be correct or incorrect, were administered to the schoolteachers; they were developed in order to assess program Purpose 1 (gaining basic knowledge about mental illness and children of parents with mental illness). The five items related to parental mental illness were lifetime prevalence of mental illness, types of mental illness, age of onset of mental illness, difficulties of living with people with mental illness, and mental health and welfare life support services. The five questions related to children were about the percentage of children with parents having mental illness, children's own awareness, counseling, impact on health, and the common roles of young carers. Some of the items were based on previous research on mental health literacy education [25, 27, 34], but the questionnaire items about children were developed originally based on the opinions of the research members since no previous research had been conducted on this topic.

#### Program goals achievement

The degree to which the participants had achieved the eight items representing the program goals was measured using a seven-point scale ranging from “not at all” (1 point) to “very well” (7 points). The following goals were set as an assessment for achieving program Purpose 1: 1) be able to describe the impact of parental mental illness on children’s lives, 2) be able to describe children’s feelings, and 3) be able to describe the significance of school for children. The following goals were set as an assessment for achieving program Purpose 2: 4) be able to recognize children in need of support. The following goals were set as an assessment for achieving program Purpose 3: 5) be able to describe how to support children, 6) be able to act appropriately toward children, and 7) be able to act appropriately in school. Professionals’ negative attitudes regarding parenting with mental illness can hamper support provision [35]. Therefore, it is important for schoolteachers to reduce the stigma against such parents and their children and have a positive attitude toward them. The final program goal is to 8) be able to support children with hope for their future.

#### Process and feasibility evaluation measures

The process and feasibility evaluation measures were as follows: program satisfaction (very satisfied, somewhat satisfied, not very satisfied, not satisfied at all); whether the participants would recommend the program to other schoolteachers (would recommend, would not recommend); length of the program (long, just right, short); what the participants learned was new (yes/no); if yes, what was impressive (open-ended text response); whether their way of thinking had changed (changed/not changed); if so, how it had changed (open-ended text response); and how they planned to act (open-ended text response).

#### Basic characteristics

The basic characteristics included age, gender, years of experience, job title, experience in special needs teaching, experience of supporting parents with mental illness, experience of supporting students with mental illness, experience of learning about mental illness, and the history of someone close, who has, or is suspected of having, a mental illness.

### Randomization and blinding

When school-based applications were received by the research office, a random number table was prepared by a person who was unrelated to the implementation process, and independent allocations were made to either the intervention or control group in the order of arrival of the applications. After the school applications were received, the same research descriptions, which were designed for individuals, were sent to both the intervention and control groups. Because of ethical considerations and the need to ensure that the participants understood the purpose of the study, we simply stated, in the research description, that the children of parents with mental illness might face challenges. After the allocation of the schools, data managers were no longer blind.

The next time any schoolteacher’s individual application form arrived at the research office, the schoolteacher was assigned to the group to which the school was assigned. The participants were guided to their allocation group after the application, and they were no longer blinded. The web surveys and the video were sent at pre-set times, using a computer research electronic data capture accumulation and management system. The quantitative data analyses were blinded to the analysis.

### Analyses

As the primary population, the full analysis set (FAS), excluding the scores of those who did not respond to T1 from all randomized participants, was used in all analyses, except process and feasibility evaluation. The per-protocol set (PPS), which excluded participants who did not respond on time, those who did not complete watching video on time in the intervention group, and those who withdrew from the study, was also defined for sensitivity analysis of the primary outcome.

Baseline characteristics are summarized as mean and standard deviation for continuous variables and frequency and proportion for categorical variables. Student’s t-test, chi-square test, and Fisher’s exact test were performed for comparisons between the intervention and control groups.

The difference in the Sense of Coping Difficulty subscale at T3 between groups was evaluated by a t-test on cluster-specific means. As a sub-analysis, a multilevel analysis with a mixed model was performed, with group as fixed effects, and school as random effects, adjusting for some baseline characteristics. We fitted a mixed model for repeated measures (MMRM), with group, time points (T1, T2, and T3) and their interactions as fixed effects, and schools and study participants as random effects.

The three actual behavioral and attitude items were compared between the groups using chi-square analysis. A multilevel analysis was then conducted using a mixed-effects logistic model.

For the 10 knowledge items, the percentage of correct answers for each item was compared between the groups using chi-square analysis or Fisher's exact test. The total number of correct answers was compared using a Wilcoxon rank-sum test. Mixed-effect logistic models were also fitted.

For program goal achievement, the medians and interquartile ranges of the scores and the total scores for each item were calculated and compared, using the Wilcoxon rank-sum test. MMRM were also fitted.

In an exploratory manner, we examined the interaction between the baseline score and intervention effects on the Sense of Coping Difficulty subscale.

The open-ended text response was analyzed qualitatively as part of a process evaluation. We created classification axes based on similarity and separated them into the smallest units whose meanings could be understood [36]. First, the first author divided the smallest units into classification axes. Then, the other researcher placed the units on the same axes. The classification of units that did not match was 5 units out of 256 units. Units that did not match were discussed by the two researchers until they matched. The number of applicable individuals—not the number of units—was counted for each classification axis.

The missing data were not imputed. A p-value of 0.05 or less was considered significant. All analyses were performed using SAS9.4 (SAS Institute Inc., Cary, NC, USA).

### Ethical procedures

This study was approved by the Ethics Committee for the Intervention Study of Osaka University Hospital (approval no. 21144; 5/10/2021). Written informed consent was obtained from all the participants. This study was conducted in accordance with the principles of the Declaration of Helsinki. This project has been registered in the Clinical Trial Registry (UMIN000045483; 14/09/2021). No adverse events were reported.

While filming one individual in the video, we asked her to decide whether she would like to have her face and name included in the video. She chose to show her face but not disclose her name. We notified the person in writing and obtained her consent for the study participants to watch the video.

## Results

### Study participants

**Fig. 1:**
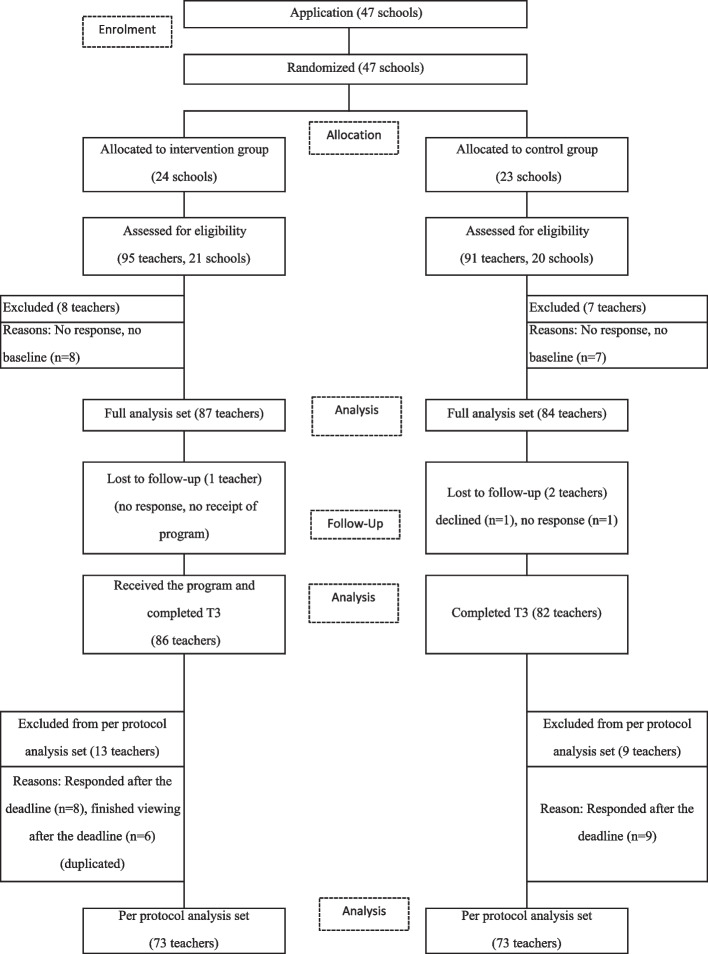
Flow of study participants

This study was conducted between October 2021 and May 2022. The flow of study participants is shown in Fig. 1. In total, 47 schools were included in this study. In order of application, the schools were divided into 24 intervention groups and 23 control groups, by cluster randomization; 95 teacher participants from 21 of the 24 schools applied for the intervention group, and 91 teacher participants from 20 of the 23 schools applied to the control group. Eight participants in the intervention group and seven in the control group could not be contacted and did not respond to baseline (T1). Therefore, 87 participants in the intervention group and 84 in the control group responded to the baseline (T1) (FAS). After T1, one participant in the intervention group could not be contacted and did not participate in the intervention program. In the control group, one participant withdrew and one participant could not be contacted.

Eighty-six participants in the intervention group and 82 in the control group completed T3. Of the 86 participants in the intervention group, 73 became PPS, excluding 8, who responded after the deadline, and 6, who watched after the deadline (with duplicates, a total of 13 participants). Of the 82 participants in the control group, 73 had PPS, excluding nine who responded after the due date.

The number of participants did not reach the originally expected number. This result was attributable to the schools’ response to COVID-19. The number of people infected with COVID-19 was declining in Japan in the fall of 2021. However, when this study began, schools reopened after the summer break; although students were able to attend school, schoolteachers were busy due to government orders to strengthen infection control measures in schools [37]. From January 2022 onwards, infections spread. More cooperation was not expected; therefore, individual entry ended in April 2022.

### Baseline characteristics of study participants

The demographics of the study participants in the intervention and control groups are shown in Table 2. More than half of the participants (69.0% in the intervention group and 56.0% in the control group) had experience of supporting parents with mental illness, but only approximately 30% (27.6% and 31.0%, respectively) had any experience in learning about mental illness. No statistically significant differences were observed between the intervention and the control groups.

**Table 2:** Baseline characteristics of study participants by group

Baseline characteristics		Intervention group (*n* = 87)		Control group (*n* = 84)		
		M n	SD (%)	M n	SD (%)	p
Age		43.6	12.2	41.1	12.3	0.188
Gender	Male	39	44.8%	35	41.7%	0.677
	Female	48	55.2%	49	58.3%	
Years of experience as a school teacher		18.8	12.3	16.0	11.3	0.123
Types of school teachers	Class teacher	36	41.4%	35	41.7%	0.715
	Managerial position teacher	24	27.6%	16	19.0%	
	Special needs teacher	9	10.3%	15	17.9%	
	Head teacher	6	6.9%	5	6.0%	
	School nurse-teacher	7	8.0%	6	7.1%	
	Nutrition teacher	1	1.1%	1	1.2%	
Experience in special needs teaching	Yes	33	37.9%	38	45.2%	0.332
	No	54	62.1%	46	54.8%	
Experience in supporting parents with mental illness	Yes	60	69.0%	47	56.0%	0.079
	No	27	31.0%	37	44.0%	
Experience in supporting students with mental illness	Yes	40	46.0%	31	36.9%	0.229
	No	47	54.0%	53	63.1%	
Experience of learning about mental illness	Yes	24	27.6%	26	31.0%	0.629
	No	63	72.4%	58	69.0%	
History of someone close to you who has or is suspected of having a mental illness	Yes	34	39.1%	39	46.4%	0.331
	No	53	60.9%	45	53.6%	

^*^P-values: t-test for continuous variables, Fisher's exact test for types of school teachers, Chi-square test for other categorical variables

### Outcome measures at endpoint (T3)

The analysis results for the Sense of Coping Difficulty subscale at T3 in the FAS and PPS are shown in Table 3. In the intervention group, the total score at T3 was significantly lower than that in the control group by t-test on the cluster-specific means on both FAS (11.6 ± 2.2 and 13.3 ± 2.9, respectively, *p* = 0.007) and PPS (11.6 ± 2.2 and 13.4 ± 3.0, respectively, *p* = 0.009), as well as multilevel analysis using a mixed model with covariates. The effect size (Cohen’s d) of the total score was –0.90 (FAS) and –0.86 (PPS), indicating large enough.

**Table 3:** Main outcomes at endpoint(T3)

		Endpoint (T3)		Effect size	Difference (I-C)			Difference (I-C)		
						t-test			mixed model	
Sense of Coping Difficulty subscale		Mean	SD	Cohen's d	Mean	95%CI	p^*^	LSmean	95%CI	p^**^
Full analysis set (FAS)	Intervention (*n* = 86)	11.6	2.2	-0.90	-1.65	[-2.81, -0.49]	0.007	-1.38	[-2.09, -0.67]	< .001
	Control (*n* = 82)	13.3	2.9							
Per-protocol analysis set (PPS)	Intervention (*n* = 73)	11.6	2.2	-0.86	-1.61	[-2.78, -0.43]	0.009	-1.44	[-2.19, -0.69]	< .001
	Control (*n* = 73)	13.4	3.0							

*I-C* Intervention and control groups

^*^t-test on the cluster-specific means

^**^ Multilevel analysis using a mixed model with baseline scores (T1), years of experience, presence or absence of experience in special needs education, presence or absence of experience in dealing with parents with mental illness, presence or absence of experience in dealing with students with mental illness, presence or absence of experience learning about mental illness, history of mental illness in their immediate family as covariates, group (intervention or control)as fixed effects, and school as random effects

### Effectiveness over time

**Fig. 2:**
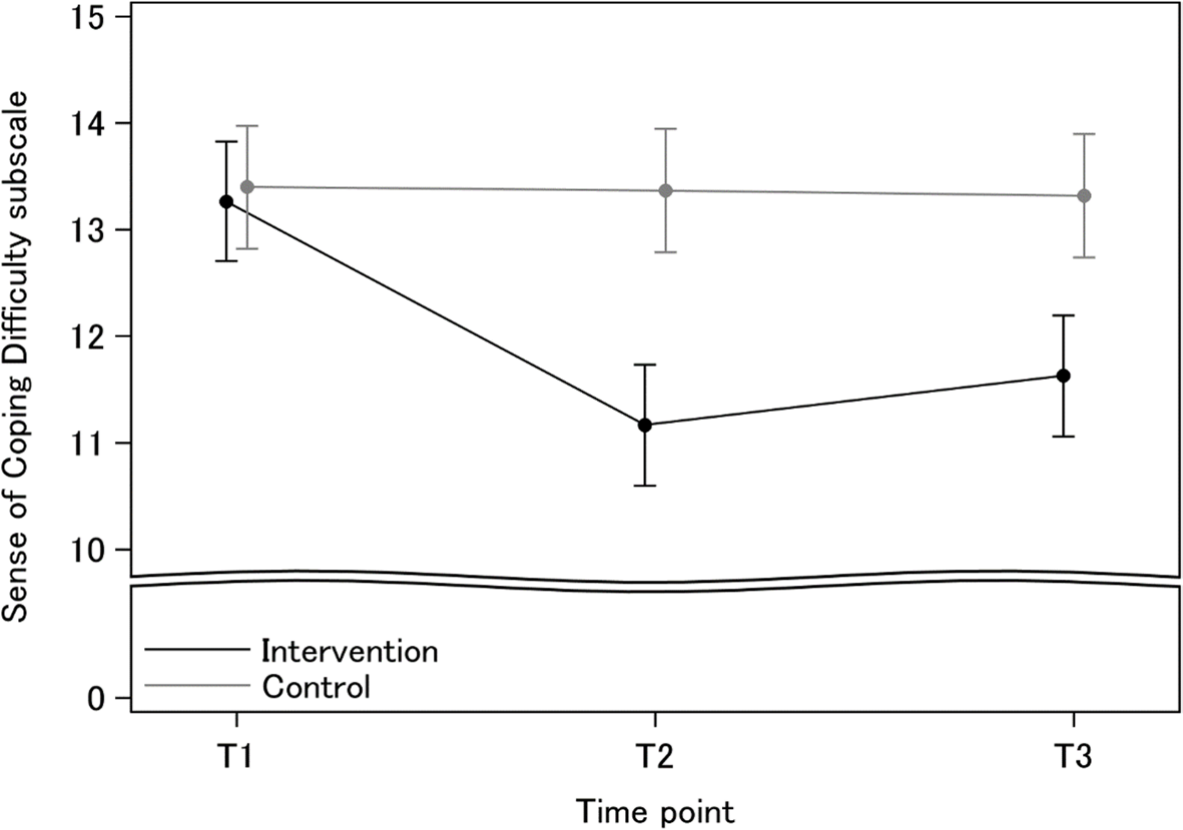
Interaction of time and intervention as T1, T2 and T3

As shown in Table 4, the analysis results of the MMRM showed that the total score on the Sense of Coping Difficulty subscale was significantly lower in the intervention group at T2 and T3 (*p* < 0.001). The least squares means estimated using the mixed model are shown in Fig. 2.

**Table 4:** Effects of intervention at T2 and T3 (FAS)

		Baseline (T1)		Post (T2)		Endpoint (T3)		group × T2	group × T3
		I,C(*n* = 87,84)		I,C(*n* = 86,82)		I,C(*n* = 86,82)			
		Mean	SD	Mean	SD	Mean	SD	p 1)	
Sense of Coping Difficulty subscale	Intervention	13.3	2.5	11.2	2.4	11.6	2.2	< .001	< .001
	Control	13.4	2.4	13.3	2.9	13.3	2.9		
Actual behaviors and attitude		n	%	n	%	n	%	p 2)	
1. Looked at children from the perspective of a child in need of support	Intervention	45	51.7	n/a	n/a	75	87.2	n/a	0.002
	Control	38	45.2	n/a	n/a	55	67.1		
2. Recognized the presence of child(ren) in need of support	Intervention	34	39.1	n/a	n/a	44	51.2	n/a	0.880
	Control	39	46.4	n/a	n/a	41	50.0		
3. Consulted other schoolteachers about child(ren) in need of support	Intervention	36	41.4	n/a	n/a	45	52.3	n/a	0.763
	Control	36	42.9	n/a	n/a	41	50.0		
Knowledge (corrected)		n	%	n	%	n	%	p 2)	
1. Mental illness affects 1 in 5 people in their lifetime	Intervention	62	71.3	78	90.7	79	91.9	< 0.001	0.020
	Control	60	71.4	56	68.3	65	79.3		
2. Alcoholism and gambling addiction are also mental illnesses	Intervention	73	83.9	86	100.0	85	98.8	0.026 **	0.614 **
	Control	74	88.1	77	93.9	80	97.6		
3. Approximately 1 in 20 children live with a parent with mental illness	Intervention	29	33.3	40	46.5	19	22.1	0.008	0.867
	Control	27	32.1	22	26.8	19	23.2		
4. Children of parents with mental illness are often aware of their parents' illnesses themselves when they reach elementary school age	Intervention	43	49.4	37	43.0	25	29.1	0.715	0.299
	Control	36	42.9	33	40.2	30	36.6		
5. Children of parents with mental illness often consult the school about family matters	Intervention	70	80.5	82	95.4	72	83.7	0.240 **	0.890
	Control	78	92.9	74	90.2	68	82.9		
6. Half of those affected by mental illness develop the illess by their mid-teens	Intervention	28	32.2	72	83.7	63	73.3	< .001	< .001
	Control	21	25.3	31	37.8	34	41.5		
7. If a parent has a mental illness, laundry and cleaning may not get done	Intervention	83	95.4	86	100.0	86	100.0	0.114 **	0.488 **
	Control	78	92.8	79	96.3	81	98.8		
8. Children whose parents have mental illness are prone to mental and physical illnesses	Intervention	83	95.4	85	98.8	84	97.7	1.000 **	1.000 **
	Control	82	97.6	81	98.8	81	98.8		
9. Performing household chores is the most common role played by children of parents with mental illness as young carers	Intervention	11	12.6	41	47.7	17	19.8	< .001	0.115
	Control	7	8.3	8	9.8	9	11.0		
10. Life support services available to people with mental illness include home visiting nursing and housekeeping services	Intervention	73	83.9	75	87.2	79	91.9	0.728	0.926
	Control	67	79.8	70	85.4	75	91.5		
		Median	IQR	Median	IQR	Median	IQR	p 3)	
Sum of 1 to 10	Intervention	6 (6–7)		8 (7–9)		7 (6–8)		< .001	0.009
	Control	6 (6–7)		6 (6–7)		6 (6–7)			
Achievement of program goals		Median	IQR	Median	IQR	Median	IQR	p 3)	
1. Be able to describe the impact of parental mental illness on the children's lives	Intervention	3	2–4	5	4–5	5	4–5	< .001	< .001
	Control	3	2–4	3	2–4	4	3–5		
2. Be able to describe the children's feelings	Intervention	3	2–4	5	4–5	5	4–5	< .001	< .001
	Control	2	2–4	3	2–4	4	2–5		
3. Be able to describe the significance of school for the children	Intervention	3	2–4	5	4–6	5	4–5	< .001	< .001
	Control	3	2–5	3	2–5	4	3–5		
4. Be able to recognize the children in need of support	Intervention	3	2–5	5	4–5	5	4–5	< .001	0.055
	Control	4	3–5	4	3–5	4	3–5		
5. Be able to describe how to support the children	Intervention	2	2–4	5	4–6	5	4–5	< .001	< .001
	Control	3	2–4	3	2–4	2	2–5		
6. Be able to act appropriately toward the children (When you notice children in need of support)	Intervention	3	2–4	5	4–5	5	4–5	< .001	< .001
	Control	3	2–4	4	2–5	4	3–5		
7. Be able to act appropriately in school (When you notice children in need of support)	Intervention	3	2–4	5	4–6	5	4–6	< .001	< .001
	Control	4	2–5	4	3–5	4	3–5		
8. Be able to support children with hope for their future	Intervention	4	3–5	5	4–6	5	4–6	< .001	0.002
	Control	4	3–5	4	3–5	5	4–5		
Sum of 1) to 8)	Intervention	24	18–32	40	34–44	39	34–42	< .001	< .001
	Control	25.5	20–34	28	21–35	31	24–39		

*IQR* interquartile range

1) a mixed model for repeated measures with group, time point (T1, T2, and T3), group, and time interactions as fixed effects, and schools and study participants as a random effect

2) chi-square analysis or Fisher's exact test(**)

3) Wilcoxon rank-sum test

Of the three actual behaviors and attitude, only “looked at children from the perspective of a child in need of support,” was significantly different at T3 (87.2% and 67.1% for the intervention and control groups, respectively, *p* = 0.002), whereas the other two behaviors were not significantly different at T3 in the chi-square test. The same was true for the mixed-effects logistic model results (not shown).

Of the 10 knowledge items, those effective up to T3 were “mental illness affects 1 in 5 people in their lifetime” (no. 1) (91.9% and 79.3% for the intervention and control groups respectively (*p* = 0.020)), “half of those affected by mental illness develop the illness by their mid-teens” (no. 6) (73.3% and 41.5%, respectively (*p* < 0.001)), and the total number of correct answers (*p* = 0.009). The same was true for the mixed-effects logistic model results (not shown).

All program goal achievement scores were not significantly different at T1 but were significantly different at T2 and T3.

Least squares means estimated by the mixed effects model.

In an exploratory manner, an analysis of the interaction between baseline and intervention effects on the Sense of Coping Difficulty subscale found a significant interaction (*p* = 0.001): for those with lower scores (lower difficulty), there was no significant difference between groups (*p* = 0.812); for those with higher scores (higher difficulty), the intervention group had a significantly greater effect than the control group (p = 0.001) as shown in Table 5.

**Table 5:** Main outcomes at endpoint(T3) by the baseline scores

		Endpoint (T3)		Difference (I-C)		
Sense of Coping Difficulty subscale		LSmean	SE	LSmean	95%CI	p^**^
Lower difficulty (*n* = 67)	Intervention (*n* = 40)	10.9	0.4	-0.14	[-1.33, 1.05]	0.812
(baseline score < 13, median)	Control (*n* = 27)	11.0	0.5			
Higher difficulty (*n* = 104)	Intervention (*n* = 46)	12.4	0.4	-1.79	[-2.83, 0.75]	0.001
(baseline score > = 13, median)	Control (*n* = 55)	14.2	0.3			

*I-C* Intervention and control groups

*FAS* Full analysis set

^**^ Multilevel analysis using a mixed model with baseline scores (T1), years of experience, presence or absence of experience in special needs education, presence or absence of experience in dealing with parents with mental illness, presence or absence of experience in dealing with students with mental illness, presence or absence of experience learning about mental illness, history of mental illness in their immediate family as covariates, group (intervention or control)as fixed effects, and school as random effects

### Process and feasibility evaluations

In the intervention group, 83 of 86 participants were "very satisfied" or "somewhat satisfied" with the program, and three were "not very satisfied." Regarding whether they would recommend the program to other schoolteachers, 85 were "willing to recommend" and 1 was "unwilling to recommend.” The length of the program was "long" (26 participants), "just right" (60 participants), and "short" (0 participants).

In terms of “what you learned was new in the program,” 82 answered “yes” and 4 answered “no.” Among those who agreed, Table 6 shows that, in descending order of importance, 21 were impressed by "the importance of staying close to children," 11 by "thoughts and feelings of children of parents with mental illness" and "the high incidence of mental illness," and 10 by "the large number of children of parents with mental illness.”

**Table 6:** Qualitative analysis of open-ended text response

Item	
Categories	n
What was particularly impressive about what was new in the program	(94)
The importance of staying close to the children	21
Thoughts and feelings of children of parents with mental illness	11
High incidence of mental illness	11
The large number of children of parents with mental illness	10
Low age of onset of mental illness	7
How parents with mental illness and their children live	6
How children of parents with mental illness live into adulthood	6
Knowledge of mental illness	5
Information on young caretakers	5
Others	12
What has changed in thinking so far after taking the program	(50)
Want to stay involved with the children	19
View of the children's background	16
Want to be actively involved with the children	5
Want to be aware of children in need of support	5
Others	5
How you plan to act	(101)
Look carefully at the children's background	22
Continue to stay close to the children	20
Try to be aware of children in need of support	19
Stay actively involved with the children	10
Have entire school respond, not just individuals	10
Share with the entire school, not just individuals	6
Create a safe environment at school	3
Share with other schoolteachers	3
Others	8

Regarding “whether your way of thinking has changed,” 60 felt that it had, while 26 felt that it had “not changed.” Of those who got “changed,” in order of preference, 19 "want to stay involved with the children," and 16 wrote, "view of the children's background.”

Regarding “how you plan to act,” the most common responses were, in descending order, "look carefully at the children's background," written by 22 participants, "continue to stay close to the children," by 20, and, "try to be aware of children in need of support,” written by 19. Related to the school's organizational response, some responded, "entire school responds, not just individuals," "share with the entire school, not just individuals," "create a safe environment at school," and "share with other schoolteachers.”

## Discussion

### Study participants

The intervention and control groups had approximately the same number of clusters and schoolteachers, with no significant baseline differences. The mean age of the study participants was 43.6 years (intervention group) and 41.1 years (control group), similar to the mean age of public elementary schoolteachers in Japan, 42.6 years [38].

Additionally, 55.2% (intervention group) and 58.3% (control group) of the study participants were female, slightly less than the 62.4% of all public schoolteachers [39]. This may be related to the fact that 27.6% (intervention group) and 19.0% (control group) of the study participants were in management positions, which is higher than the figure for public elementary schoolteachers as a whole (9.1%) [39]. It can be assumed that more administrators participated in this study because school principals were asked to participate on a school-by-school basis.

The experience of working with parents with mental illness was common to more than half of the participants, but only about 30% had learned about mental illness. Among elementary schoolteachers who resigned because of illness, 69.1% resigned because of mental illness [38], thus indicating that mental illness is a familiar illness for schoolteachers. It is believed that some of the participants in the study participated because they were concerned about mental illness but were experiencing difficulties in dealing with it; they had no experience in learning about it.

### Effectiveness of the program

The program was effective in reducing schoolteachers’ difficulty in supporting children of parents with mental illness. The effect sizes were as large as –0.90 (FAS) and –0.86 (PPS). Goal attainment was also effective for over one month. Few studies on mental health interventions for schoolteachers have been conducted using RCTs [21]. To date, there have been no reports on programs to help schoolteachers support children of parents with mental illness (such as this current program). Therefore, this is valuable as an evidence-based program.

Although exploratory in nature, this program was found to be particularly effective for those with higher scores (higher difficulty in support) on the Sense of Coping Difficulty subscale. The program included what children appreciated about their schoolteachers, such as staying close to them and listening to them. In the analysis of the open-ended text response, many schoolteachers planned to continue to stay close to the children. Staying close to children does not require schoolteachers to develop any specialized skills in dealing with mental illness. This may have given schoolteachers the confidence to provide support.

Knowledge was effective up to the T3; the correct response rate of “mental illness affects 1 in 5 people in their lifetime (no. 1),” and “half of those affected by mental illness develop the illness by their mid-teens (no. 6).” These items were objective measures of the onset timing and probability of onset. However, no significant effects were found for items not expressed numerically (no. 4, 5, 7, 8, 10). Many previous programs on schoolteachers’ mental health literacy have also been effective in increasing knowledge [21]. However, a previous study has found effects on knowledge regarding the probability of onset but not so much on the knowledge that is not expressed numerically (e.g., symptoms) [27]. Therefore, knowledge that cannot be demonstrated with objective numbers may be less likely to show effects.

For actual behaviors and attitude, only “looked at children from the perspective of a child in need of support” had a significant effect in the past month, whereas “recognized the presence of a child(ren) in need of support” and “consulted within the school about a child(ren) in need of support” had no significant effect. There were significant effects on goal attainment related to confidence at the behavioral level (nos. 4–7). In the analysis of the open-ended text response, “the importance of staying close to children” was the most common impression (21 participants), and “continue to stay close to children” was also the second most common action plan (20 participants). The “view of the children's background” also changed their outlook (16 participants), and they were willing to “look carefully at the children's background” (22 participants). Therefore, it is thought that, although they were able to look at the children from the viewpoint of children in need of support, this was not reflected in changes in their behavior (e.g., recognizing the existence of the children or consulting with the children at school). One possible reason is that the child(ren) in need of support could not be detected in a month. Therefore, a long-term evaluation may help schoolteachers recognize such a child(ren). Another possible reason could be that children are wary of their surroundings because they do not want their parents to be known. A total of 55% of adult children of parents with mental illness reported that they did not show signs that those around them could recognize when they were in elementary school [16]. Therefore, schoolteachers must not only recognize such children but also make it easy for them to consult with schoolteachers. Screening sheets written by the children themselves have been developed to identify young carers [40], and these may make it a little easier to identify children in need of support.

All program goal achievement scores remained effective up to one month later. We believe that the effect took hold for one month, not only due to the program content but also because of how it was structured. While developing this program, we considered the current lack of social awareness regarding the existence of children with parents having mental illness. Referring to the TTM, the program included self-re-evaluation, and we asked the following questions before providing new knowledge, “Have you ever met a child like this?” Baseline participants’ information indicated that more than half of them had experience working with parents having mental illness. Therefore, it is assumed that the participants recalled children they had met in the past and reflected on their own responses at that time. Furthermore, after watching the independent adult in the video, they may have thought about the aftermath of their own support. It is believed that they could become more confident in their support and more willing to provide support with hope. Though this program involved a short 30-min video, we believe that the structure of this program was also effective in that its effect lasted for one month.

### Program feasibility and adaptation to practice

Although Japanese schoolteachers are very busy, 168 of the 171 baseline participants remained in the study until the end. This may have been due to the short duration of the program (30 min) and the easy participation in the e-learning format, which was not restricted to any location and allowed them to choose their own locations. The length of the program was “long” (26 participants), “just right” (60 participants), and “short” (none). Therefore, although a shorter length would have been more desirable, the current one was considered acceptable. Thirty minutes would be sufficient to set up a training session with additional group work after watching the video. As mentioned in the open-ended text response section, it is sometimes difficult to solve problems unless they are shared and handled by the entire school. The need for an approach that involves the entire school has been pointed out in a previous study [20]. In a national survey of Japanese elementary schools, 80% of schools were found to have meetings to discuss support for children with concerns, but only 13.5% had screening meetings to identify children in need of support [18]. A screening meeting might allow schoolteachers to share information with other schoolteachers, staff, and administrators and help them identify children in need of support; this may be expected to be more effective compared to the efforts of only one schoolteacher who has identified a child in need of support. This program can be used as a training program within the school, and administrators can organize a screening meeting to identify children in need of support or to formulate other organizational responses.

## Study limitations

This study had several limitations. First, the program assessed the understanding and behaviors of individual schoolteachers but not the organizational response of the school as a whole. There were opinions that in order to identify and support children, not only the efforts of individual schoolteachers, but also the whole school approach is needed. Therefore, in the future, this program should be shared with the entire school and evaluated from an organizational perspective as well. Second, it was assumed that the study participants had some interest in the issue, and those who had no interest in the issue at all would not have participated in this study. Therefore, we do not know whether this program is effective for those who have no interest in the issue. Third, mental health conditions involve a wide spectrum of conditions. This program does not cover all mental illnesses.

## Conclusion

We developed a 30-min video-based e-learning program to help elementary schoolteachers support children of parents with mental illness and evaluated its effectiveness using a school-based cluster randomized controlled trial. It was observed that it was significantly effective, over one month, in reducing difficulties in supporting children.

## Data Availability

The materials for this program are openly available (https://kageyamaresearch.wixsite.com/watashikoko). These data are also available from the corresponding author upon request. The datasets generated and analyzed during the current study are not publicly available owing to confidentiality concerns, but are available from the corresponding author on reasonable request, subject to the approval of the institutional ethics review committee.

## Abbreviations

ANOVA: Analysis of variance
FAS: Full analysis set
ICC: Intraclass correlation coefficients
MMRM: Mixed model for repeated measures
PPS: Per-protocol analysis set
RCT: Randomized controlled trial
TTM: Trans-theoretical Model
